# Total Synthesis of Talarolide A and *atrop*-Talarolide A: Hydroxamate H-Bond Bridge Stabilization of Cyclic Peptide Conformers Invokes Non-Canonical Atropisomerism

**DOI:** 10.3390/md22100454

**Published:** 2024-10-03

**Authors:** Waleed M. Hussein, Yuxuan Zhu, Angela A. Salim, Robert J. Capon

**Affiliations:** Institute for Molecular Bioscience, The University of Queensland, St. Lucia, QLD 4072, Australia; yuxuan.zhu1@uq.net.au (Y.Z.); a.salim@uq.edu.au (A.A.S.)

**Keywords:** talarolide A, total synthesis, atropisomerism, cyclic peptide, conformer stabilization, hydroxamate H-bond bridge, marine-derived fungus, *Talaromyces*, natural product

## Abstract

The first total synthesis of the Australian marine tunicate fungus-derived cyclic peptide talarolide A (**1**) has confirmed the structure previously proposed on the basis of spectroscopic and chemical analyses and re-affirmed the importance of the unique hydroxamate H-bond bridge in ring conformer stabilization. The unexpected co-synthesis of *atrop*-talarolide A (**8**) revealed, for the first time, that hydroxamate H-bond bridging in the talarolide framework invokes non-canonical atropisomerism and that talarolides A (**1**), C (**3**), and D (**4**) all exist naturally as atropisomers. These discoveries raise the intriguing prospect that comparable functionalisation of other cyclic peptides, including those with commercial value, could provide ready access to new “unnatural atropisomeric” chemical space, with new and/or improved chemical and biological properties.

## 1. Introduction

Cyclic peptides feature prominently in the natural products landscape. This is particularly true of those produced by microbes (bacteria and fungi), with noteworthy examples exhibiting valuable biological properties that have prompted the development of a range of commercial products, including antibacterials (e.g., daptomycin, vancomycin, colistin, and gramacidin), antifungals (e.g., echinocandins), immunosuppressives (e.g., cyclosporin), and antiparasitics (e.g., emodepside). Many microbial cyclic peptides are biosynthetically derived from non-ribosomal peptide synthetases (NRPS), often with heavy modification of the essential amino acids, and can include additional structural units through either mixed biosynthesis and/or tailoring enzymes. Structural modifications can extend to stereodiversity (l and d configurations), as well as C, N and O alkylation and acylation, halogenation and glycosylation, and can be inclusive of other biosynthetic units (e.g., alkaloids, lipids, terpenes, polyketides, etc.) and cyclic peptide ring sizes. For example, in our own studies into the chemistry of Australian marine-derived microbes, we have reported on cyclic peptides inclusive of lipo-cyclodidepsipeptide acremolides from a Tasmanian estuarine *Acremonium* sp. [1]; cyclopentapeptide cotteslosins from a Western Australian beach sand-derived *Aspergillus* sp. [2]; glyco-cyclohexadepsipeptide-polyketide mollemycin A from a north Queensland marine sediment-derived *Streptomyces* sp. [3]; anthelmintic octapeptide surugamides from various Australian marine and terrestrial *Streptomyces* spp. [4]; and cycloheptapeptide hydroxamate talarolides from a Queensland marine tunicate-derived fungus *Talaromyces* sp. [5,6]. The latter are particularly interesting in that their study highlighted both the challenges encountered in the structure elucidation of natural cyclic peptides that are often only accessible in very small quantities, while at the same time revealing a novel functionality (hydroxamate H-bond bridging) that at least in the case of the talarolides stabilized cyclic peptide conformers.

**Figure 1:**
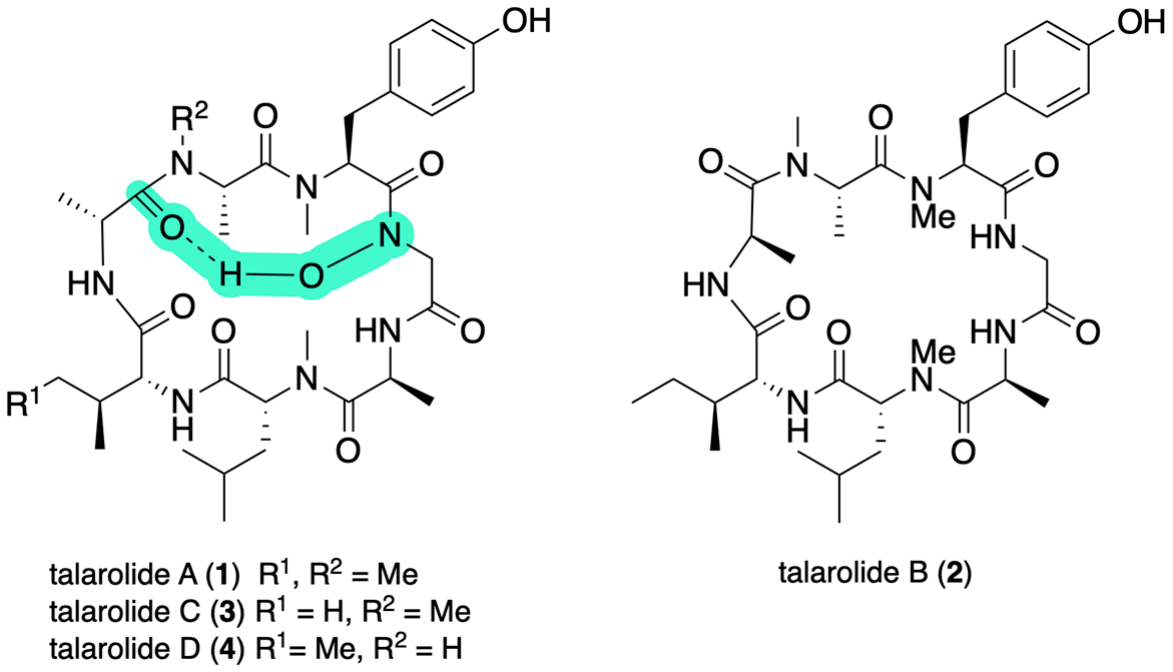
Talarolides A–D (**1**–**4**). Color highlight—hydroxamate H-bond bridge (green).

Talarolide A (**1**) was first reported in 2017 as only the second known example of a cyclic peptide featuring a hydroxamate moiety and the first to be documented with a ring-spanning hydroxamate H-bond bridge [5]. A subsequent 2023 report on the re-isolation of **1** from the same marine-derived fungus allowed for the acquisition of higher-quality spectroscopic data and the design and implementation of refined analytical procedures, which prompted a structural revision of **1** [6]. In addition to providing 3D modelling in support of the conformational impact of the hydroxamate H-bridge, this latter study also enabled the isolation and structure elucidation of the co-metabolite talarolides B–D (**3**–**4**) (Figure 1). Significantly, where **1** and **3**–**4** featured a strong hydroxamate H-bond bridge and existed as single well-defined conformers, the related metabolite **2** which lacked an *N*-OH moiety and was hence incapable of forming a hydroxamate H-bond bridge existed as two equilibrating conformers. Based on these observations, we speculated that the inclusion of hydroxamate H-bond bridges within cyclic peptides may be a means to restrict conformational flexibility. In an effort to explore this concept further, and add further evidence for structural assignment, in this report, we describe the first total synthesis of talarolide A (**1**) and reveal factors that influence the formation of the hydroxamate H-bond bridge.

## 2. Results

**Figure 2:**
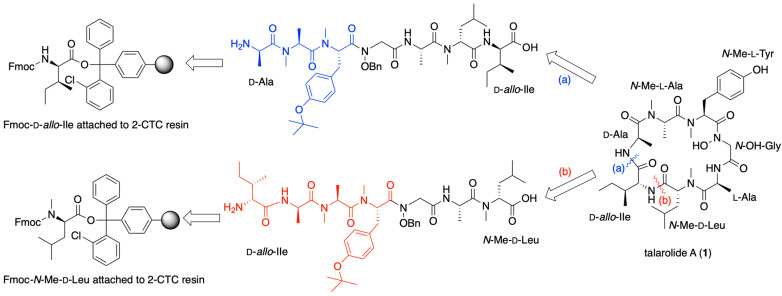
Retrosynthesis of talarolide A (1). (a) Cleavage between D-*allo*-Ile and D-Ala leads to three residues after coupling of *N*-benzyloxy Gly, highlighted in blue; (b) Cleavage between *N*-Me-D-Leu and D-*allo*-Ile leads to four residues after coupling of *N*-benzyloxy Gly, highlighted in red.

In designing the synthesis of talarolide A (**1**), we envisaged a traditional solid-phase approach to a suitably protected linear precursor, which upon release from the resin would undergo a sequence of deprotection and cyclization to arrive at the desired product. In selecting a point of disconnection (i.e., which amide bond to be opened to arrive at a linear precursor), we avoided *N*-Me and *N*-OH amide linkages, reasoning that these would be adversely impacted by steric hindrance and/or reduced nucleophilicity at the terminal amine [7,8,9,10]. We also excluded starting with *N*-acyl-*N*-benzyloxy Gly at the C-terminus (i.e., ester bond with 2-CTC resin), anticipating that the benzyloxy moiety would be unstable to repeated solid-phase base treatments (i.e., DIPEA and piperidine). The two potential remaining disconnection sites were the amide bond linking (a) d-*allo*-Ile and d-Ala, and (b) *N*-Me-d-Leu and d-*allo*-Ile (Figure 2). We selected the former as it required adding only three residues after coupling with *N*-benzyloxy Gly. In contrast, the alternate disconnection would require adding four residues, which could compromise the stability of the benzyloxy moiety.

### 2.1. Linear Peptide (Scheme 1)

**Scheme 1:**
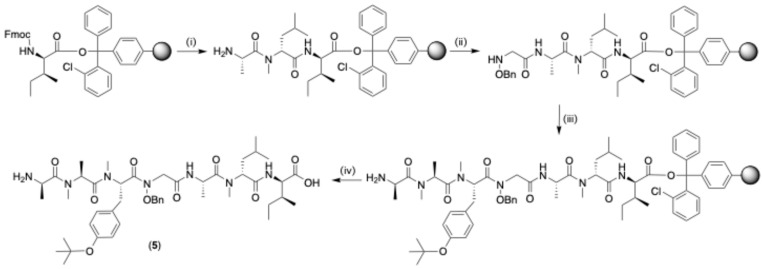
Synthesis of the protected linear peptide **5**. (i) Iterative Fmoc-SPPS [20% piperidine in DMF, RT, 5 and 10 min; Fmoc-AA-OH (3.2 equiv.), HATU (3 equiv.), DIPEA (6 equiv.), DMF, RT, 2 × 30 min, or 2 × 3 h for coupling of Fmoc-AA-OH to *N*-Me-AA-OH], (ii) coupling of *O*-benzyloxy Gly [Br-CH_2_COOH (4 equiv.), DIC (4.5 equiv.), HOBT(4.5 equiv.), DMF, RT, 2 × 1.5 h; *O*-benzyl hydroxyl amine hydrochloride (3 equiv.), DIPEA (6 equiv.), DMF, RT, 2 × 1 day]; (iii) iterative Fmoc-SPPS [Fmoc-*N*-Me-Tyr(*tert*-Bu)-OH (3.2 equiv.), HATU (3 equiv.), DIPEA (6 equiv.), DMF, RT, 2 × 1 day; 20% piperidine in DMF, RT, 5 and 10 min; Fmoc-AA-OH (3.2 equiv.), HATU (3 equiv.), DIPEA (6 equiv.), DMF, RT, 2 × 3 h for the coupling of Fmoc-AA-OH to *N*-Me-AA-OH]; (iv) 20% HFIP/DCM, RT, 3 × 20 min.

Synthesis of the protected linear peptide **5** proceeded as follows: 2-CTC resin coupled to Fmoc-d-*allo*-Ile was subjected to (i) sequential chain elongation with *N*-Me-d-Leu then l-Ala, followed by (ii) acylation of the terminal amine with bromo acetic acid, followed by substitution of Br with *O*-benzyl hydroxylamine, then (iii) sequential chain elongation with *O*-t-Bu protected *N*-Me-l-Tyr, then *N*-Me-l-Ala and then d-Ala, and finally (iv) cleavage from the resin to yield the doubly protected linear peptide precursor **5** in a 9% overall yield (Scheme 1).

### 2.2. Cyclization (Scheme 2)

**Scheme 2:**
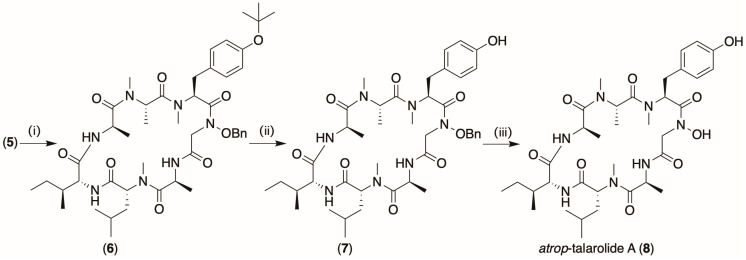
Cyclization prior to deprotection to yield *atrop*-talarolide A (**8**). (i) HATU, HOBT, and collidine 14 h, (ii) 90% formic acid 40 min, and (iii) Pd/charcoal, H_2_ gas (1.5 h).

In our first attempt to convert **5** to **1,** we elected to cyclize **5** to **6**, before carrying out sequential deprotection of the *O*-t-Bu moiety to yield **7** and the *O*-Bn moiety to yield **8** (3 mg, 37%, overall yield from **5** to **8**) (Scheme 2). Significantly, while **8** proved to be isomeric with **1** and shared an identical MS/MS fragmentation profile (Figure S1), and hence amino acid sequence, it did not co-elute on UPLC (Figure S2). In addition, there were differences in the 1D and 2D NMR (DMSO-*d*_6_) data for **1** versus **8** (Figures S3 and S4), perhaps the most telling of which was the lack of ROESY correlations so characteristic for the hydroxamate H-bond bridge in **1** (Figures S5 and S6). Collectively, these observations, together with the fact that exposure to heat and/or mild acid conditions did not encourage **8** to transform to **1**, led us to conclude the former was an alternate non-interconverting conformational isomer (i.e., non-canonical atropisomer) of **1**, designated *atrop*-talarolide A (**8**), where the *N*-OH moiety was oriented external to the cyclic peptide ring. This unexpected outcome prompted reconsideration of the sequence of reactions needed to transform **5** into **1**.

### 2.3. Cyclization (Scheme 3)

Building on our first attempt (Scheme 1), it was reasoned that cyclization prior to deprotection positioned the *N*-OBn moiety external to the peptide ring, such that even once deprotected, it was incapable of folding inward to form the hydroxamate H-bond bridge, yielding instead the non-canonical atropisomer, *atrop*-talarolide A (**8**). If correct, it seemed likely that the synthesis of **1** from **5** might be better achieved by deprotection prior to cyclization. To this end, **5** was subjected to sequential deprotection of the *O*-t-Bu moiety to yield **9** and *O*-Bn moiety to yield **10**, with subsequent cyclization yielding a non-equilibrating 1:2 mixture of two isomeric cyclic peptides, identified as **8** and **1**. This mixture was subsequently resolved via HPLC fractionation, with putative synthetic **1** shown to be identical to the natural product **1** via comparison of their NMR (DMSO-*d*_6_) (Figures S10 and S11) and MS/MS (Figure S12) spectra and via UPLC co-elution (Figure S13).

**Scheme 3:**
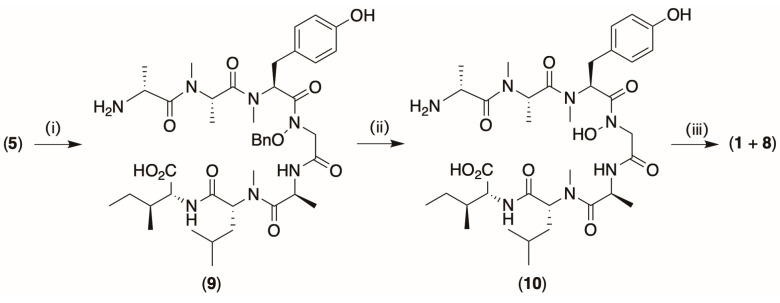
Deprotection prior to cyclization to yield talarolide A (**1**) and *atrop*-talarolide A (**8**). (i) Ninety percent formic acid 40 min, (ii) Pd/charcoal, H_2_ gas (1.5 h), and (iii) HATU, HOBT, and collidine, 14 h.

## 3. Discussion

The successful synthesis of talarolide A (**1**) as outlined above provides valuable supporting evidence for the proposed structure and reinforces the presence of the unique hydroxamate H-bond bridge. Unexpectedly, it also reveals a relationship between hydroxamate H-bond bridging and atropisomerism that is deserving of comment.

Atropisomerism is a phenomenon well known among natural products and is most commonly encountered as hindered rotation about a single axis of chirality, leading to individual atropisomers that can be isolated and which possess different 3D structures, as well as chemical and biological properties (much as is the case with stereoisomers about a chiral center). Individual atropisomers can be stable and incapable of interconversion if the barrier to rotation is high enough, or can be capable of slow interconversion with or without stimuli (i.e., heating, pH, or light). While typically referred to as “atropisomers”, the more correct terminology to describe the relationship between isomers that are interconvertible by rotation about a single axis of chirality is “canonical atropisomers”.

Atropisomerism can also involve hindered concerted rotation about a multitude of bonds. Such atropisomers are distinct from the canonical atropisomers noted above and are designated as “non-canonical atropisomers”. Some noteworthy examples of non-canonical atropisomer cyclic peptides include the clinically important glycopeptide antibiotic vancomycin [11], and the emerging new Gram-negative antibacterial darobactin A [12,13]. Interestingly, such non-canonical atropisomerism typically results from the incorporation of one or more non-peptide bond axis of chirality (i.e., diphenyl ether for vancomycin, and tryptophan ether for darobectin). To the best of our knowledge, atropisomerism has remained unknown among NRPS cyclic peptides lacking additional conformationally constrained carbo or heterocyclic systems—with the non-canonical atropisomer pair of talarolide A (**1**) and *atrop*-talarolide A (**8**) being first-in-class.

## 4. Materials and Methods

### 4.1. General Experiment

All chemicals used in this study were analytical grade. *N*-α-fluorenylmethoxycarbonyl (Fmoc)-l-amino acids, 2-chlorotrityl chloride resin (2-CTC, resin substitution: 1.55 mmol/g), collidine, hydroxybenzotriazole (HOBt), acetonitrile (MeCN), dichloromethane (DCM), high-performance liquid chromatography (HPLC) grade methanol (MeOH), *N*,*N*-dimethylformamide (DMF), *N*,*N*-diisopropylcarbodiimide (DIC), *N*,*N*-diisopropylethylamine (DIPEA), formic acid, and piperidine were purchased from Merck (Bayswater, VIC, Australia). Fmoc-d-alanine-OH and *O*-benzylhydroxylamine hydrochloride were purchased from Fluorochem Ltd. (Graphite Way, Hadfield, UK). Fmoc-*N*-Me-d-Leu-OH, Fmoc-d-*allo*-Ile-OH, and Fmoc-*N*-Me-Tyr(^t^Bu)-OH were purchased from Combi-Blocks, Inc (San Diego, CA, USA). 1-[Bis(dimethylamino)methylene]-1*H*-1,2,3-triazolo[4,5-b]pyridinium 3-oxid hexafluorophosphate (HATU), and hexafluoro-2-propanol (HFIP) were purchased from Novachem (Heidelberg West, VIC, Australia). Nuclear magnetic resonance (NMR) spectra were acquired on a Bruker Avance 600 MHz spectrometer with either a 5 mm PASEL ^1^H/D-^13^C Z-Gradient probe or 5 mm CPTCI ^1^H/^19^F-^13^C/^15^N/DZ-Gradient cryoprobe. The spectra were acquired at 25 °C in DMSO-*d*_6_ and referenced to residual signals (δ_H_ 2.50 and δ_C_ 39.5 ppm) in deuterated solvent. High-resolution ESIMS measurements were obtained on a Bruker micrOTOF mass spectrometer via direct infusion in MeCN at 3 μL/min using sodium formate clusters as an internal calibrant. UPLC-QTOF analysis was performed on a UPLC-QTOF instrument comprising an Agilent (Mulgrave, VIC, Australia) 1290 Infinity II UPLC (Agilent Zorbax SB-C_8_ RRHD 1.8 μm, 2.1 × 50 mm column, gradient elution at 0.417 mL/min over 2.50 min from 90% H_2_O/MeCN to 100% MeCN with a constant 0.1% formic acid/MeCN modifier) coupled to an Agilent 6545 QTOF mass detector. Liquid chromatography–diode array–mass spectrometry (HPLC-DAD-MS) data were acquired on an Agilent 1260 series separation module equipped with an Agilent G6125B series single quad mass detector and diode array detector (Agilent Poroshell 120 SB-C_8_ 2.7 μm, 3.0 × 150 mm column, gradient elution at 0.8 mL/min over 6.25 min from 90% H_2_O/MeCN to 100% MeCN with a constant 0.05% formic acid/MeCN modifier). Ultra-high performance liquid chromatograms (UPLCs) were obtained on an Agilent 1290 infinity UPLC system composed of a 1290 infinity binary pump, a thermostat, an autosampler, and a diode array detector (Agilent Zorbax SB-C_8_ RRHD 1.8 μm, 2.1 × 50 mm column, gradient elution at 0.417 mL/min over 2.50 min from 90% H_2_O/MeCN to 100% MeCN with a constant 0.01% TFA/MeCN modifier). Preparative and semi-preparative HPLCs were performed using an Agilent 1100 Series diode array and/or multiple wavelength detectors and an Agilent 1100 Series fraction collector. Chromatography solvents were of HPLC grade supplied by Merck and filtered/degassed through a 0.45 μm polytetrafluoroethylene (PTFE) membrane prior to use. Deuterated solvents were purchased from Novachem (Heidelberg West, VIC, Australia).

### 4.2. Synthesis of Linear Protected Peptide ***5***

*Coupling of the first amino acid*. A solution of 2-CTC resin (129 mg, 0.2 mmol scale, substitution: 1.55 mmol/g) swelled for 20 min in dry DCM (2 mL) was treated with a solution of Fmoc-d-*allo*-Ile (1.2 eq.) and DIPEA (88 µL, 0.5 mmol, 2.5 eq.) in dry DCM (2 mL), and stirred for 2 h, after which the resin was filtered and treated with MeOH (200 µL) for 15 min to cap the resin. The coupled resin was sequentially washed with dry DCM (5 × 1 min), 1:1 DCM/MeOH (5 × 1 min), and MeOH (2 × 1 min).

*Elongation of peptide sequence*. Amino acid activation was achieved by dissolving a Fmoc-amino acid (0.64 mmol, 3.2 eq.) in a 0.4 M HATU/DMF solution (1.5 mL, 0.6 mmol, 3.0 eq.) followed by the addition of DIPEA (210 μL, 1.2 mmol, 6.0 eq.). The coupling cycle consisted of Fmoc deprotection with 20% piperidine in DMF (twice, 5 and 10 min) and a 5 min DMF flow-wash followed by coupling with preactivated Fmoc-amino acid (3.2 eq.) over 2 × 30 min or 2 × 3 h for coupling of Fmoc-amino acids to sterically hindered *N*-Me-amino acids. Upon the completion of synthesis, the resin was washed with DMF, DCM, and MeOH and then dried under a vacuum desiccator.

*Coupling of O-benzyloxy Gly to the resin*. First, a mixture of bromoacetic acid (111 mg, 0.8 mmol, 4 eq) and DIC (139 μL, 114 mg, 0.9 mmol, 4.5 eq) was dissolved in DMF (0.2 mL) and maintained for 25 min. Then, the supernatant was added to the resin, followed by a solution of HOBT (122 mg, 0.9 mmol, 4.5 eq.) in DMF (0.5 mL) and mixed for 1.5 h. This process was repeated once. After sequential washing with DMF (3 × 1 min), DCM:DMF 3:1 (3 × 1 min), and DCM (3 × 1 min), a solution of *O*-benzyl hydroxyl amine hydrochloride (96 mg, 0.6 mmol, 3 eq.) and DIPEA (210 μL, 1.2 mmol, 6 eq.) in DMF (0.3 mL) was added to the resin (2 × 1 day). The resin was washed with DMF (3 × 1 min), DCM:DMF 3:1 (3 × 1 min), and DCM (3 × 1 min). The next Fmoc-amino acid was coupled using HATU and DIPEA (2 × 1 d) followed by acetylation using a mixture of 90% DMF, 5% DIPEA, and 5% acetic anhydride solution (2 mL × 2 × 10 min).

*Cleavage of linear protected peptide (***5***)*. After swelling the 2-CTC resin for 20 min in dry DCM (2 mL), the resin was mixed with 20% hexafluoro-2-propanol (HFIP)/DCM (2 mL × 3 × 20 min and the combined filtrate concentrated in vacuo to give the crude protected linear peptide that was purified via semi-preparative HPLC (Agilent Zorbax Eclipse XDB-C8 5 μm, 9.4 × 250 mm column, with a 20 min gradient elution at 3 mL/min from 90% H_2_O/MeCN to 100% MeCN with an isocratic 0.01% TFA/MeCN modifier). After lyophilization, the protected linear peptide **5** was obtained as an amorphous powder (15 mg, 9%), with purity confirmed using HPLC-DAD-MS (*t*_R_ = 5.1 min, Figure S15): ESI(+)MS *m*/*z* 882 [M+H]^+^.

### 4.3. Cyclization via Scheme 2 to Yield ***8***

*Cyclization of linear protected peptide* **5** *to yield* **6**. 

A solution of the linear protected peptide 5 (0.5 mg/mL) in DMF (20 mL, 10 mg, 0.011 mmol) was stirred vigorously and treated via dropwise addition over 30 min with a mixture of 0.4 M HATU (82.5 μL, 0.033 mmol, 3 eq.), hydroxybenzotriazole (HOBT) (5 mg, 0.033 mmol, 3 eq.), and collidine (4.5 µL, 4 mg, 0.033 mmol, 3 eq.) in DMF (2 mL). After 14 h, HPLC-DAD-MS analysis of the mixture showed that cyclization was complete. The DMF was evaporated, and the residue was dissolved in MeCN (1 mL), filtered (0.45 µm), and purified via semi-preparative HPLC (Agilent Zorbax Eclipse XDB-C_8_ 5 μm, 9.4 × 250 mm column, gradient elution at 3 mL/min over 20 min from 90% H_2_O/MeCN to 100% MeCN with an isocratic 0.01% TFA/MeCN modifier). After lyophilization, the protected cyclic peptide 6 was obtained as an amorphous powder with purity confirmed using HPLC-DAD-MS (*t*_R_ = 3.1 min, Figure S16): ESI(+)MS *m*/*z* 684 [M+H]^+^.

*Cyclic peptide deprotection—removal of the Tyr O-t-butyl group from* **6** *to yield* **7**.

The protected cyclic peptide **6** was stirred for 40 min in an aqueous solution of 90% formic acid (3 mL) after which a mixture of 50/50 H_2_O/MeCN (3 mL) was added, and the mixture was lyophilized to yield the semi-protected cyclic peptide **7** with the product confirmed using HPLC-DAD-MS (*t*_R_ = 5.8 min, Figure S17): ESI(+)MS *m*/*z* 808 [M+H]^+^.

*Cyclic peptide deprotection—removal of the Gly N-hydroxy benzyl group from* **7** *to yield* **8**.

The semi-protected cyclic peptide **7** (3.5 mg) in MeOH (5 mL) was stirred with palladium/charcoal under hydrogen gas (1.5 h), after which the mixture was filtered to yield the unnatural conformational isomer designated as;

*atrop*-talarolide A (**8**) (3 mg, 37% over 3 steps) with the purity confirmed using UPLC analysis (*t*_R_ = 1.88, Figure S2): 1D and 2D NMR (DMSO-*d*_6_); see Figures S3–S5; HRESIMS *m*/*z* 740.3984 [M + Na]^+^ (calcd for C_35_H_55_ N_7_NaO_9_, 740.3953) (Figure S7).

### 4.4. Cyclization via Scheme 3 to Yield ***1*** and ***8***

*Linear peptide deprotection—removal of the Tyr O-t-butyl group from* **5** *to yield* **9**.

The protected linear peptide **5** in aqueous solution of 90% formic acid (3 mL) was stirred for 40 min, after which a mixture of 50/50 H_2_O/MeCN (3 mL) was added, and the mixture was lyophilized to yield the semi-protected linear peptide **9** with the product confirmed using HPLC-DAD-MS (*t*_R_ = 4.5 min, Figure S18): ESI(+)MS *m*/*z* 826 [M+H]^+^.

*Linear peptide deprotection—removal of the Gly N-hydroxy benzyl group from* **9** *to yield* **10**.

The semi-protected linear peptide **9** in MeOH (5 mL) was stirred with palladium/charcoal under hydrogen gas (1.5 h), after which the mixture was filtered to yield the unprotected linear peptide **10** with the product confirmed using HPLC-DAD-MS (*t*_R_ = 4.1 min, Figure S19): ESI(+)MS *m*/*z* 736 [M+H]^+^.

*Cyclization of linear unprotected peptide* **10** *to yield* **1** *and* **8**.

A vigorously stirred solution of the linear unprotected peptide **10** (0.5 mg/mL) in DMF (6 mL, 3 mg, 0.004 mmol) was treated (dropwise addition over 30 min) with a mixture of 0.4 M HATU (30 μL, 0.012 mmol, 3 eq.), hydroxybenzotriazole (HOBT) (2 mg, 0.012 mmol, 3 eq.), and collidine (2 µL, 2 mg, 0.012 mmol, 3 eq.) in DMF (2 mL). After 14 h, HPLC-DAD-MS analysis of the mixture showed the cyclization was complete. The DMF was evaporated to yield a 2:1 mixture of two isomeric cyclic peptides, which were dissolved in MeCN (1 mL), filtered (0.45 µm), and purified via semi-preparative HPLC (Agilent Zorbax Eclipse XDB-C_8_ 5 μm, 9.4 × 250 mm column, gradient elution at 3 mL/min over 20 min from 90% H_2_O/MeCN to 100% MeCN with an isocratic 0.01% TFA/MeCN modifier) to yield;

talarolide A (**1**) (1 mg, 24% over 3 steps): UPLC-DAD (**1**, *t*_R_ = 1.85 min), (Figure S13) 1D and 2D NMR (DMSO-*d*_6_); see Figures S10 and S11; HRESIMS *m*/*z* 740.3973 [M + Na]^+^ (calcd for C_35_H_55_N_7_NaO_9_, 740.3953) (Figure S14).

*atrop*-talarolide A (**8**) (0.5 mg, 12% over 3 steps) with purity confirmed using UPLC (**8**, *t*_R_ = 1.88 min), Figure S8): HRESIMS *m*/*z* 740.3975 [M + Na]^+^ (calcd for C_35_H_55_N_7_NaO_9_, 740.3953) (Figure S9).

## 5. Conclusions

The successful total synthesis of talarolide A (**1**) as outlined above provided independent evidence for the structure of this marine natural product, consistent with that previously proposed on the basis of detailed spectroscopic and chemical analyses. Significantly, the unanticipated encounter with *atrop*-talaraolide A (**8**) revealed, for the first time, that natural talarolide A (**1**), and by inference talarolides C–D (**3**–**4**), exist as non-canonical atropisomers defined by a common hydroxamate H-bond bridge, with the latter also inducing stable cyclic peptide conformers. In contrast, the unnatural *atrop*-talarolide A (**8**) features an *N*-OH moiety oriented external to the cyclic peptide ring, that lacks a hydroxamate H-bond bridge, and exists as a mixture of conformers. Also of note, the atropisomers **1** and **8** are non-equilibrating, suggestive of a high barrier to rotation.

The unexpected discovery of atropisomerism in the talarolide scaffold raises an interesting philosophical question—*how does one recognize the presence of atropisomerism in natural products where only a single atropisomer is available?* It is true that if atropisomerism is the only form of chirality in a molecule, it can be readily detected by measuring a specific rotation, however, this option is not available for molecules that contain one or more additional chiral centers and that exhibit a specific rotation irrespective of whether they are or are not capable of atropisomerism. It is also true that atropisomerism can in some cases be predicted in molecules that feature a very obvious and highly hindered axes of chirality, but this is generally less evident in non-canonical atropisomers (i.e., simple NRPS cyclic peptides without covalent cross ring linkage such as seen in talarolide A). For example, talarolide A (**1**) exhibits chirality due to amino acid residues, with biosynthetic modifications limited to *N*-methylation and hydroxylation and stereo-inversion. When originally isolated, there was no reason to suspect atropisomerism, until our synthetic efforts revealed the unnatural atropisomer **8**.

The knowledge that talarolide atropisomerism is linked to the unique hydroxamate H-bond bridge raises the prospect that the inclusion of a similar *N*-OH moiety and associated H-bond bridge in other cyclic peptides may induce atropisomerism and/or conformational stabilization. That *N*-OH regiochemistry can be varied around a cyclic peptide (i.e., which amide bond is modified to a hydroxamate) could open up access to a new dimension of cyclic peptide chemical space featuring defined but different atropisomers with stable well-defined conformations. If applied to commercially important cyclic peptides, it is intriguing to speculate if this strategy would impart new and/or improved chemical and biological properties.

## Data Availability

NMR and mass spectrometric data are available upon request.

## Supplementary Materials

The following supporting information can be downloaded at: https://www.mdpi.com/article/10.3390/md22100454/s1. Spectroscopic and chemical characterization of **1** and **5**–**10**.
