# Supplementary material for: Total Synthesis of Talarolide A and atrop-Talarolide A: Hydroxamate H-Bond Bridge Stabilization of Cyclic Peptide Conformers Invokes Non-Canonical Atropisomerism
Source: Mar Drugs. 2024 Oct 3;22(10):454. doi: 10.3390/md22100454 (PMC11509828; doi:10.3390/md22100454)
Supplement: Supplementary file 1 [file marinedrugs-22-00454-s001.zip › marinedrugs-3230355-supplementary.pdf]

Supporting Information

Total Synthesis of Talarolide A and *atrop*-Talarolide A: Hydroxamate H-Bond Bridge Stabilization of Cyclic Peptide Conformers Invokes Non-Canonical Atropisomerism.

Waleed M. Hussein*, Yuxuan Zhu, Angela A. Salim and Robert J. Capon*

Institute for Molecular Bioscience, The University of Queensland, St. Lucia, QLD, 4072, Australia

* Correspondence: RJC: r.capon@uq.edu.au; WMH: w.hussein@uq.edu.au.

1. Spectroscopic characterisation of *atrop*-talarolide A (8)

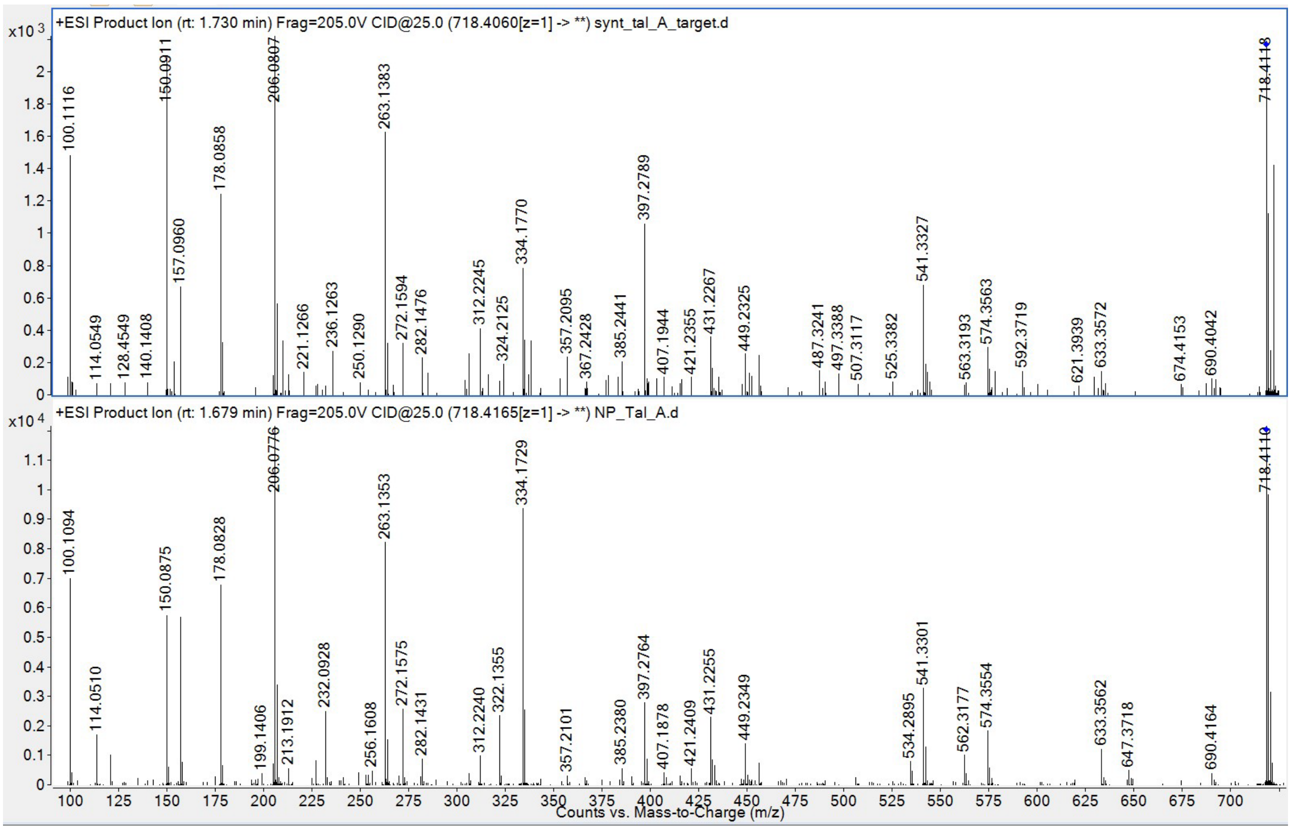

Figure S1. MSMS chromatograms of *atrop*-talarolide A (**8**) (top) and natural talarolide A (**1**) (bottom)

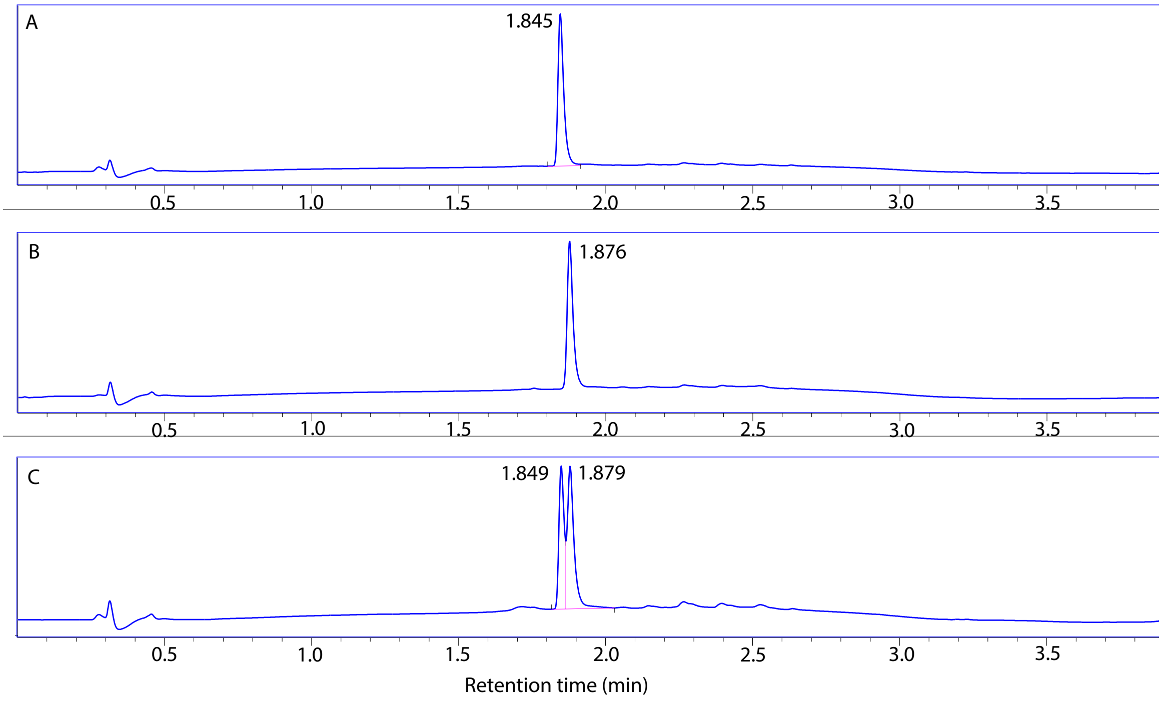

Figure S2. UPLC-DAD (210 nm) chromatograms of (A) natural talarolide A (**1**), (B) *atrop*-talarolide A (**8**), and (C) co-injection of **1** and **8**.

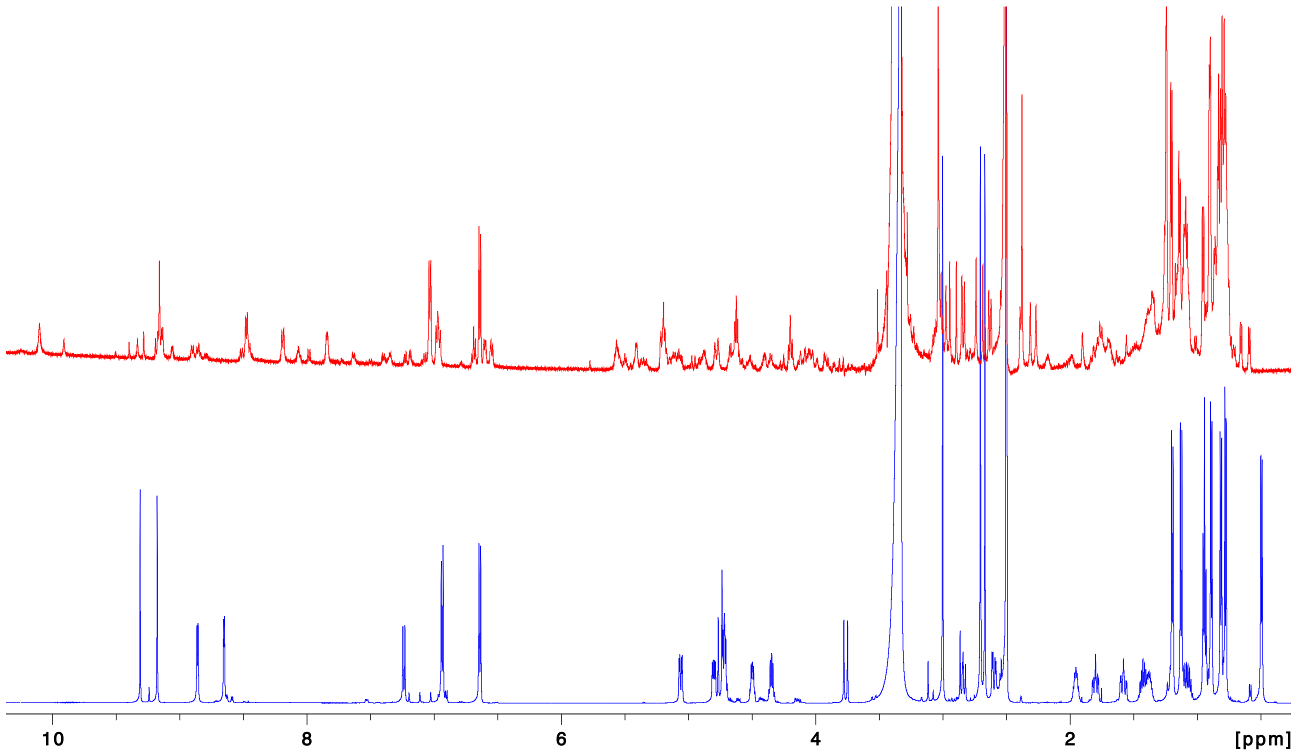

Figure S3. Overlay of ^{1}H NMR (600 MHz, DMSO-d_6) spectra of *atrop*-talarolide A (**8**) (red) and natural talarolide A (**1**) (blue).

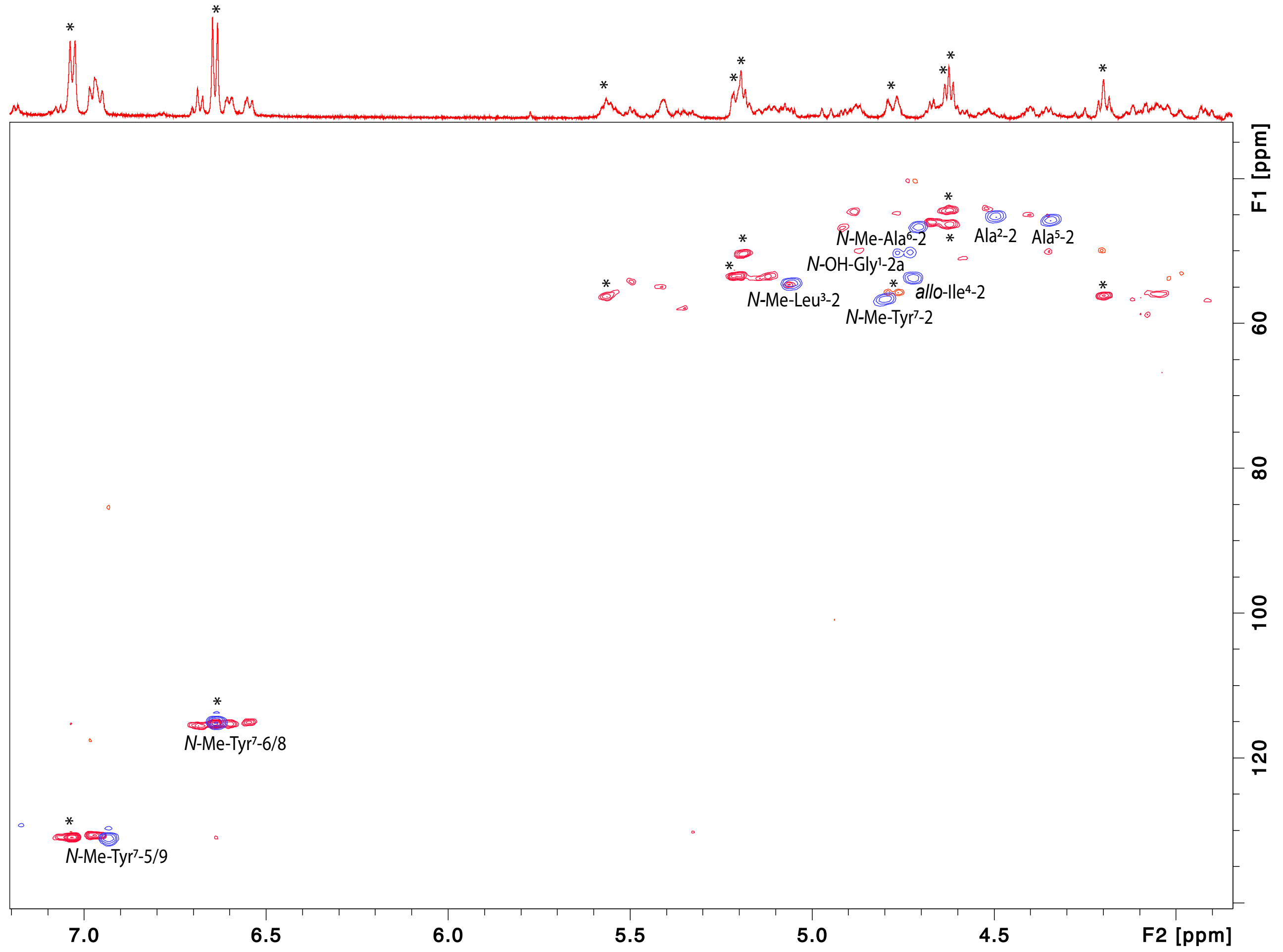

Figure S4. Overlay of HSQC (600 MHz, DMSO-d_6) spectra of *atrop*-talarolide A (**8**) (red) and natural talarolide A (**1**) (blue) - annotated, * indicates signals from the major conformer of *atrop*-talarolide A (**8**).

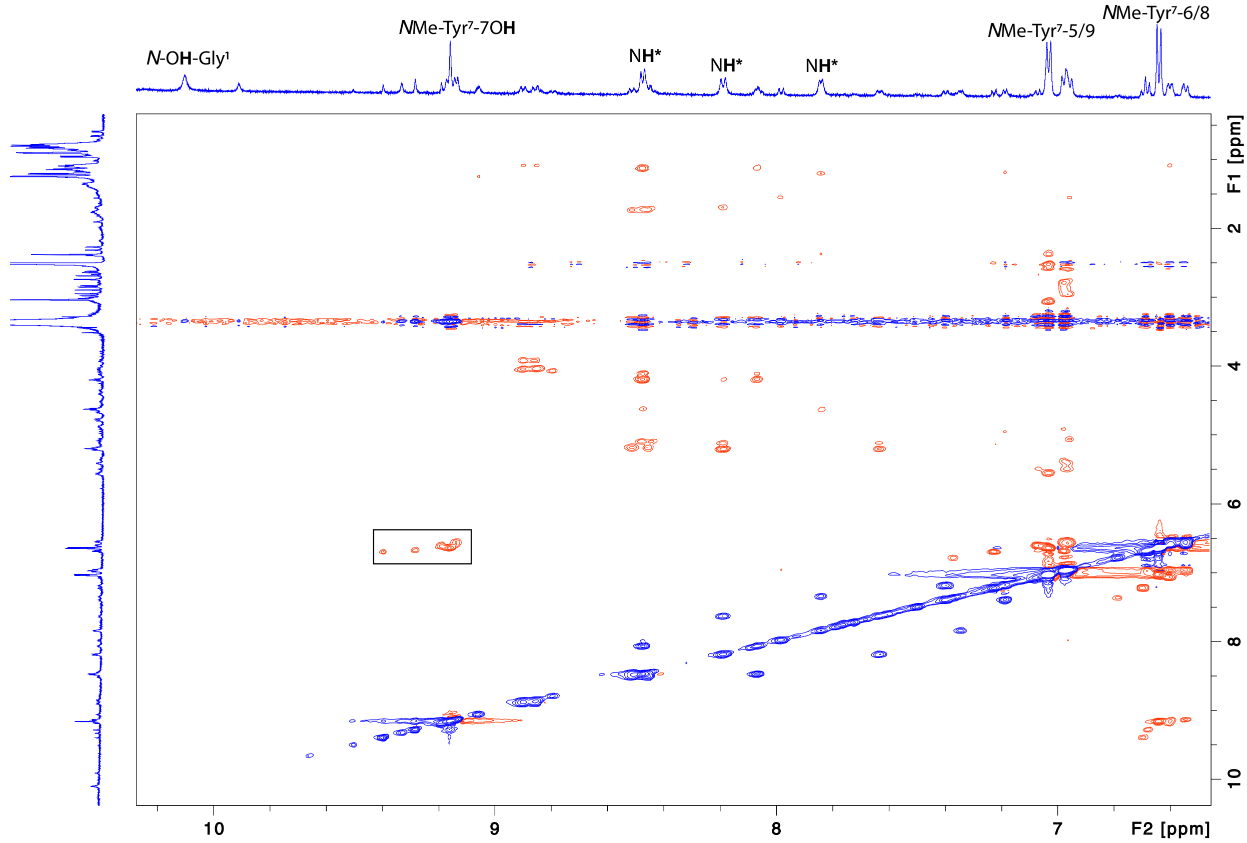

Figure S5. ROESY NMR (600 MHz, DMSO-d_6) spectrum of *atrop*-talarolide A (**8**). There were no observable ROESY correlations from *N*-O**H**-Gly. Black box highlighted ROESY correlations between *N*Me-Tyr^7-7O**H** and *N*Me-Tyr^7-6/8. *NH are not assigned to a particular amino acid residue.

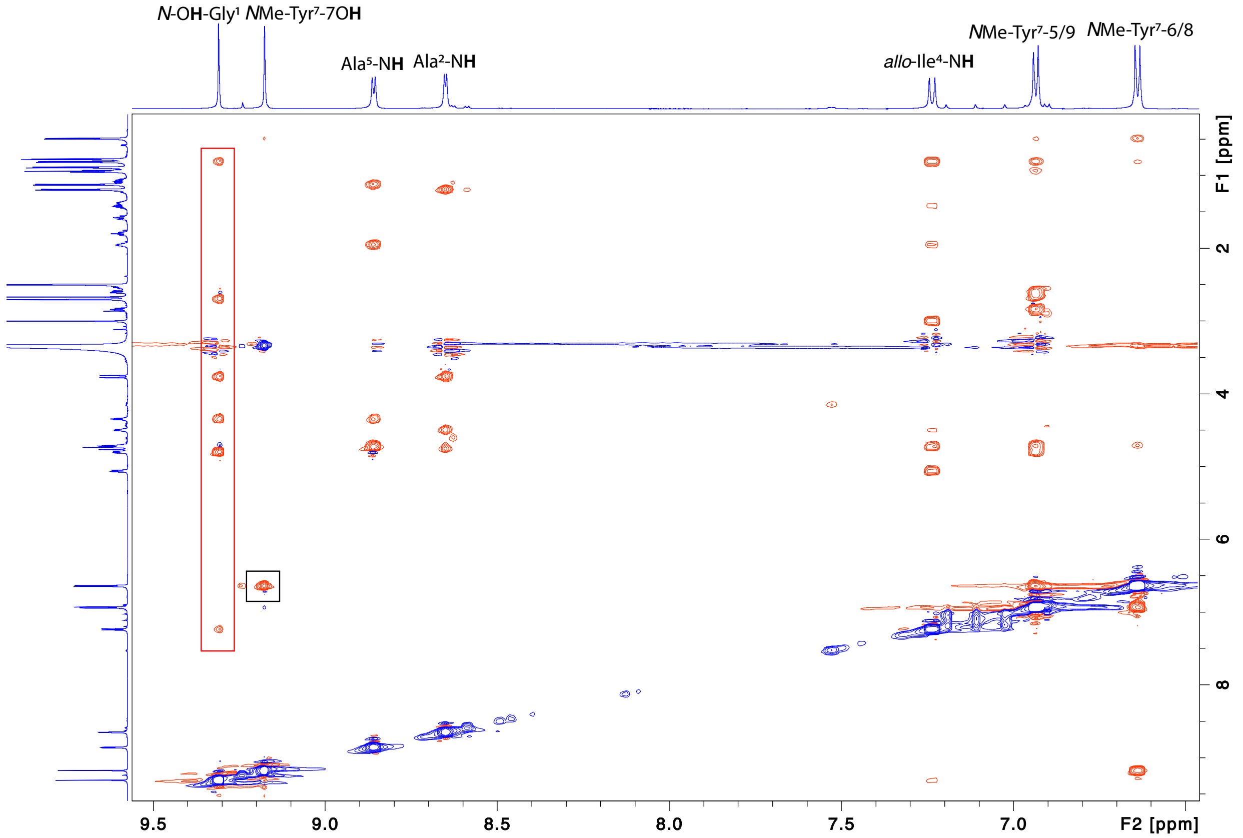

Figure S6. ROESY NMR (600 MHz, DMSO-d_6) spectrum of natural talarolide A (**1**). Red box highlighted ROESY correlations between *N*-O**H**-Gly to protons of other amino acid residues in close proximity. Black box highlighted ROESY correlations between *N*Me-Tyr^7-7O**H** and *N*Me-Tyr^7-6/8.

Mass Spectrum Molecular Formula Report

Analysis Info

Analysis Name	D:\Data\a.salim\Tal_A_synt.d	Acquisition Date	5/24/2023 12:15:24 PM	
Method	tune-med_AP.m	Operator	a.salim	
Sample Name		Instrument / Ser#	micrOTOF	213750.00232
Comment				

Acquisition Parameter

Source Type	ESI	Ion Polarity	Positive	Set Nebulizer	0.8 Bar
Focus	Not active			Set Dry Heater	180 °C
Scan Begin	100 m/z	Set Capillary	4500 V	Set Dry Gas	5.0 l/min
Scan End	1000 m/z	Set End Plate Offset	-500 V	Set Divert Valve	Source

Generate Molecular Formula Parameter

Formula, min.		
Formula, max.		
Measured m/z	Tolerance	Charge
Check Valence	Minimum	Maximum
Nirogen Rule	Electron Configuration	
Filter H/C Ratio	Minimum	Maximum
Estimate Carbon		

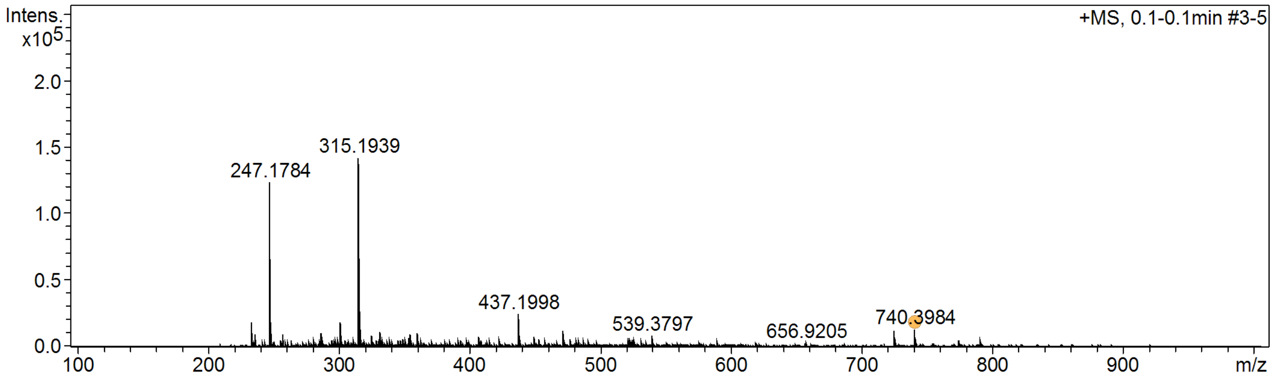

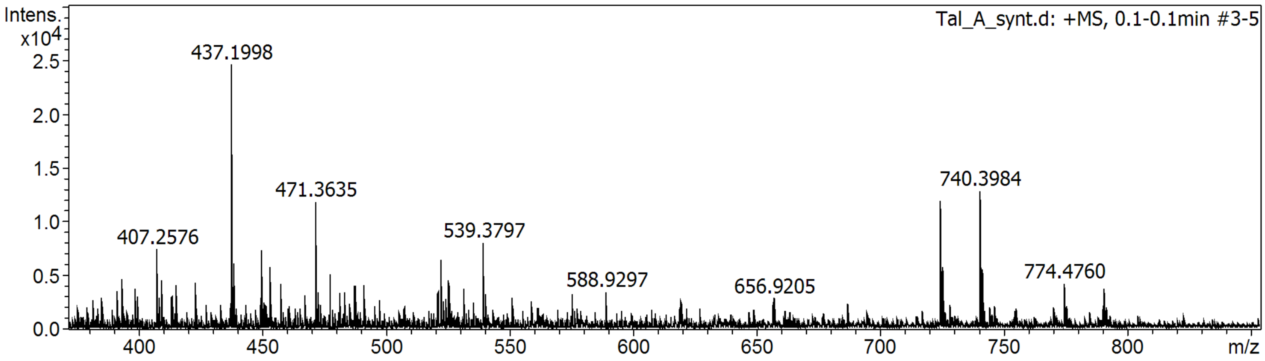

Meas. m/z	#	Ion Formula	m/z	err [ppm]	mSigma	# Sigma	Score	rdb	e⁻ Conf	N-Rule
740.3984	1	C35H55N7NaO9	740.3953	4.2	26.4	1	100.00	11.5	even	ok

Figure S7. HRMS measurement for *atrop*-talarolide A (**8**) obtained from Scheme 2.

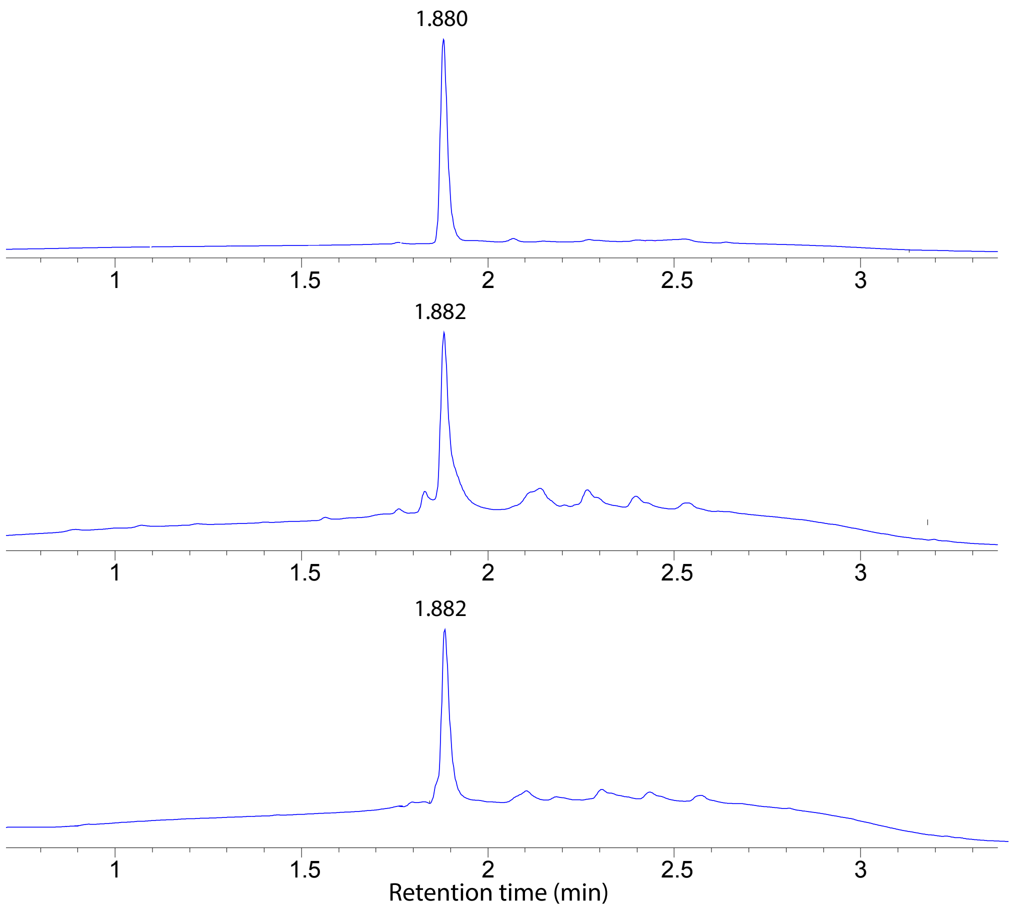

Figure S8. UPLC-DAD (210 nm) chromatograms of *atrop*-talarolide A (**8**) obtained from (A) Scheme 2 and (B) Scheme 3 and (C) co-injection of both.

Mass Spectrum Molecular Formula Report

Analysis Info

Analysis Name	D:\Data\a.salim\Tal_A_synt_2.d	Acquisition Date	10/21/2023 12:50:20 PM
Method	tune-med_AP.m	Operator	a.salim
Sample Name		Instrument / Ser#	micrOTOF 213750.00232
Comment			

Acquisition Parameter

Source Type	ESI	Ion Polarity	Positive	Set Nebulizer	0.8 Bar
Focus	Not active			Set Dry Heater	180 °C
Scan Begin	100 m/z	Set Capillary	4500 V	Set Dry Gas	5.0 l/min
Scan End	1000 m/z	Set End Plate Offset	-500 V	Set Divert Valve	Source

Generate Molecular Formula Parameter

Formula, min.		
Formula, max.		
Measured m/z	Tolerance	Charge
Check Valence	Minimum	Maximum
Nirogen Rule	Electron Configuration	
Filter H/C Ratio	Minimum	Maximum
Estimate Carbon		

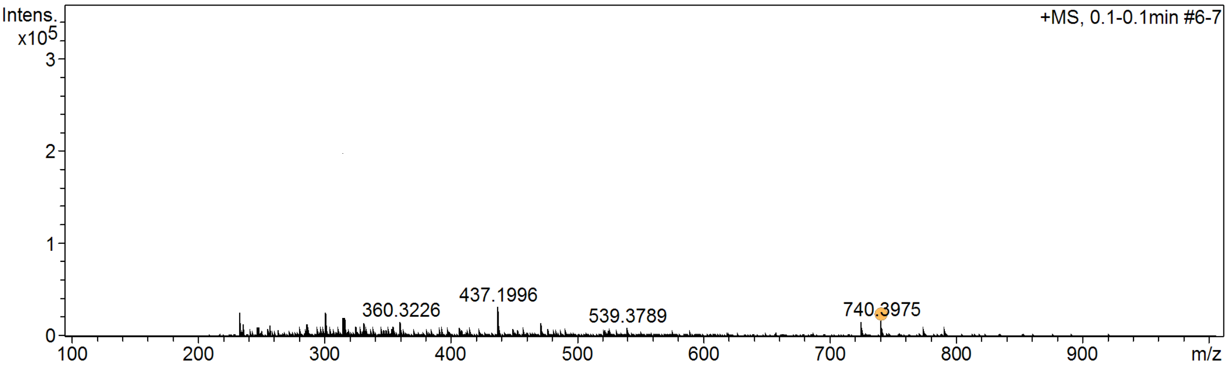

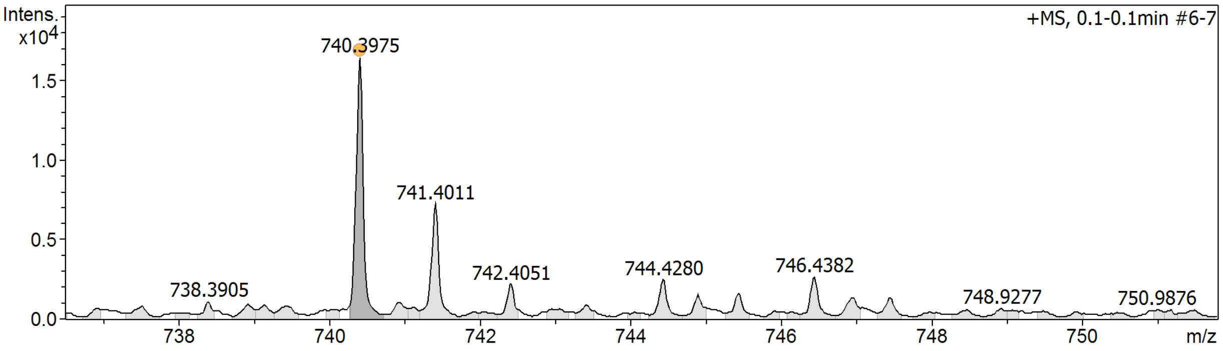

Meas. m/z	#	Ion Formula	m/z	err [ppm]	mSigma	# Sigma	Score	rdb	e⁻ Conf	N-Rule
740.3975	1	C35H55N7NaO9	740.3953	-2.9	27.4	1	100.00	11.5	even	ok

Figure S9. HRMS measurement for *atrop*-talarolide A (**8**) obtained from Scheme 3

2. Spectroscopic characterisation of synthetic talarolide A (1)

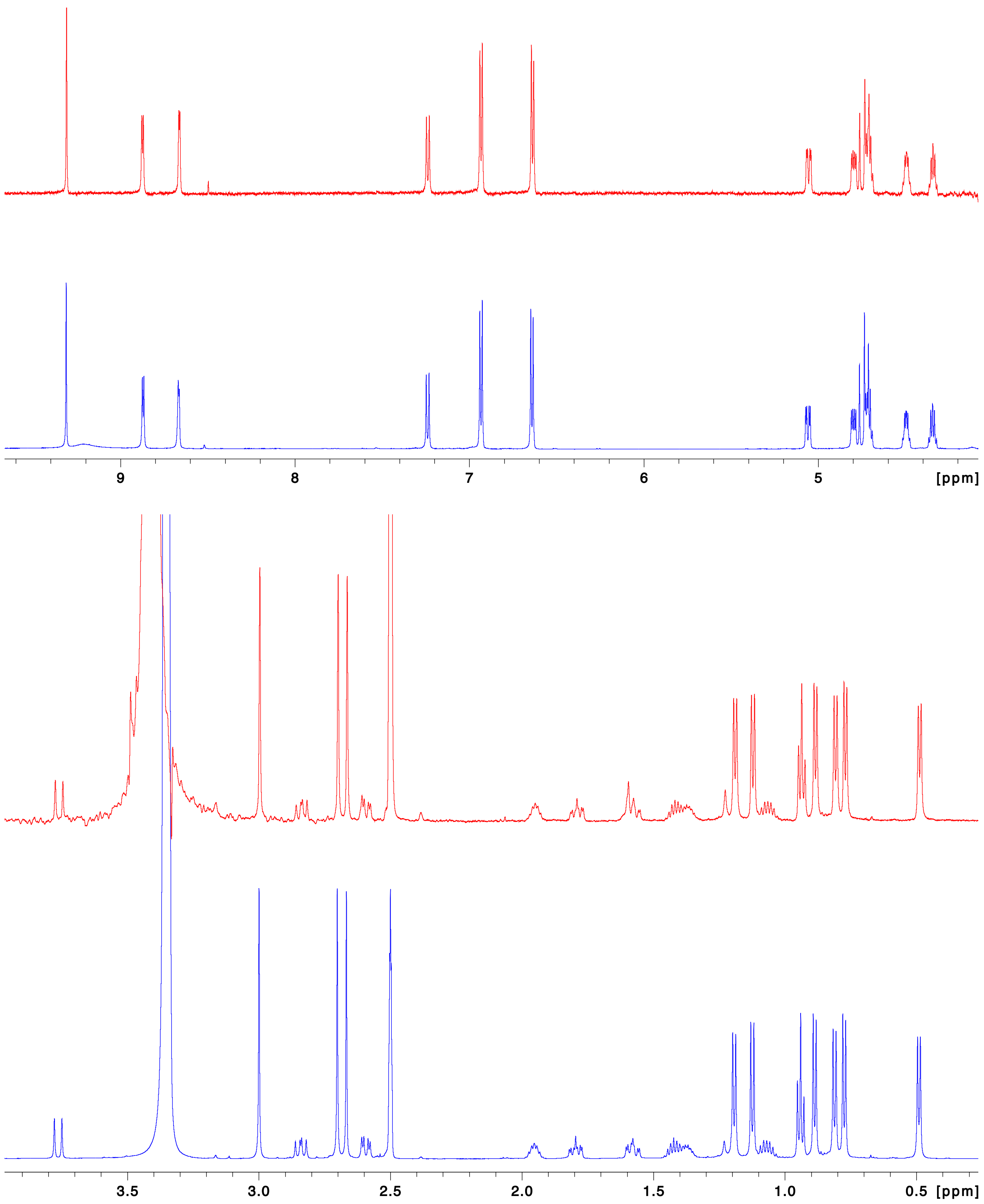

Figure S10. Overlay of ^{1}H NMR (600 MHz, DMSO-d_6) spectra of synthetic (red) and natural (blue) talarolide A.

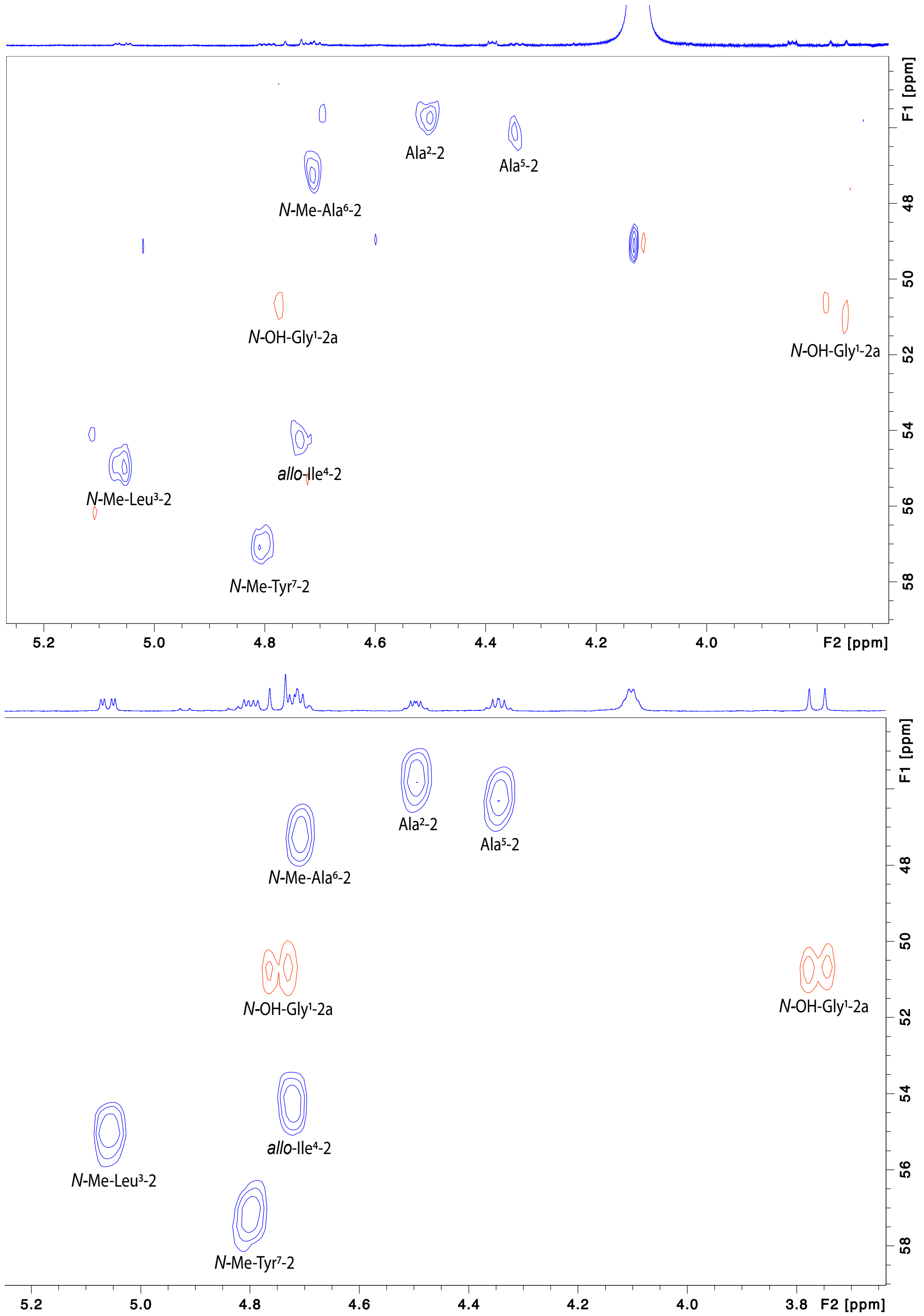

Figure S11. Expanded HSQC (600 MHz, DMSO-d_6) spectra of synthetic (top) and natural (bottom) talarolide A showing the alpha protons region.

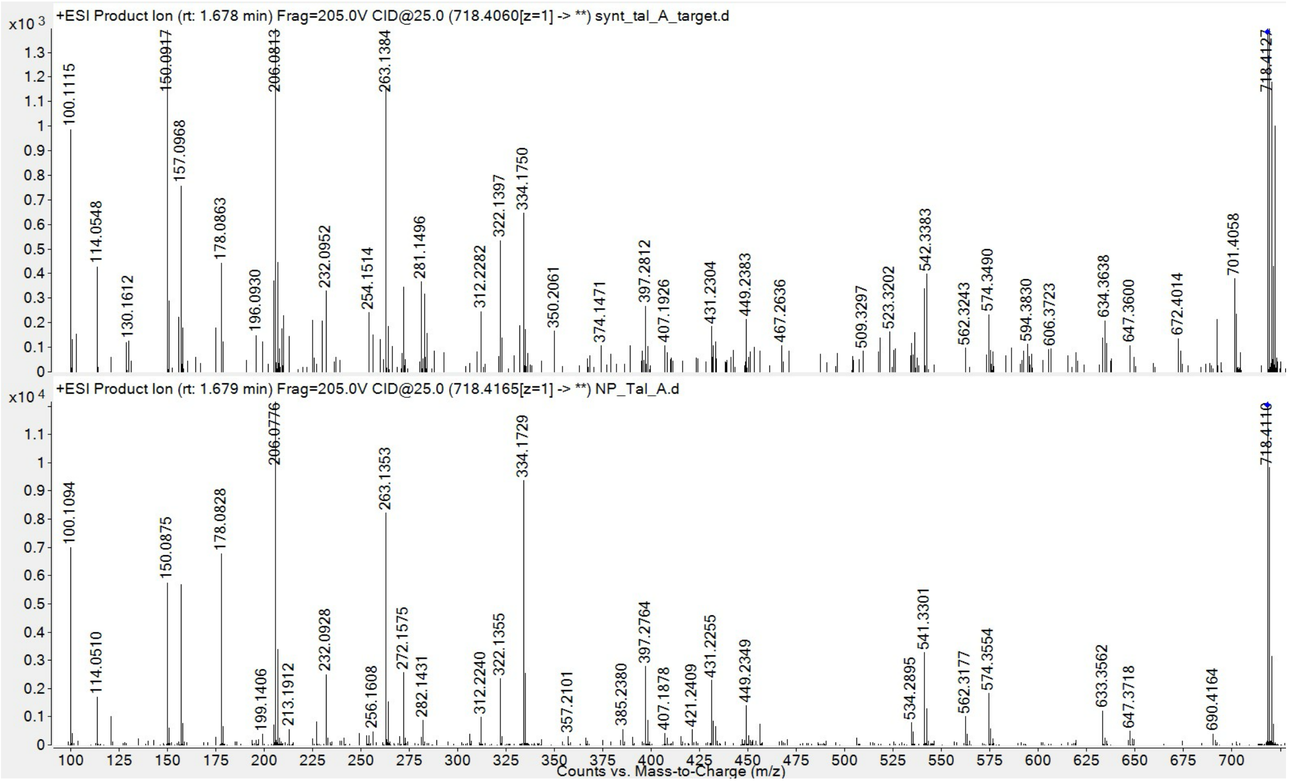

Figure S12. MSMS chromatograms of synthetic (top) and natural (bottom) talarolide A (**1**)

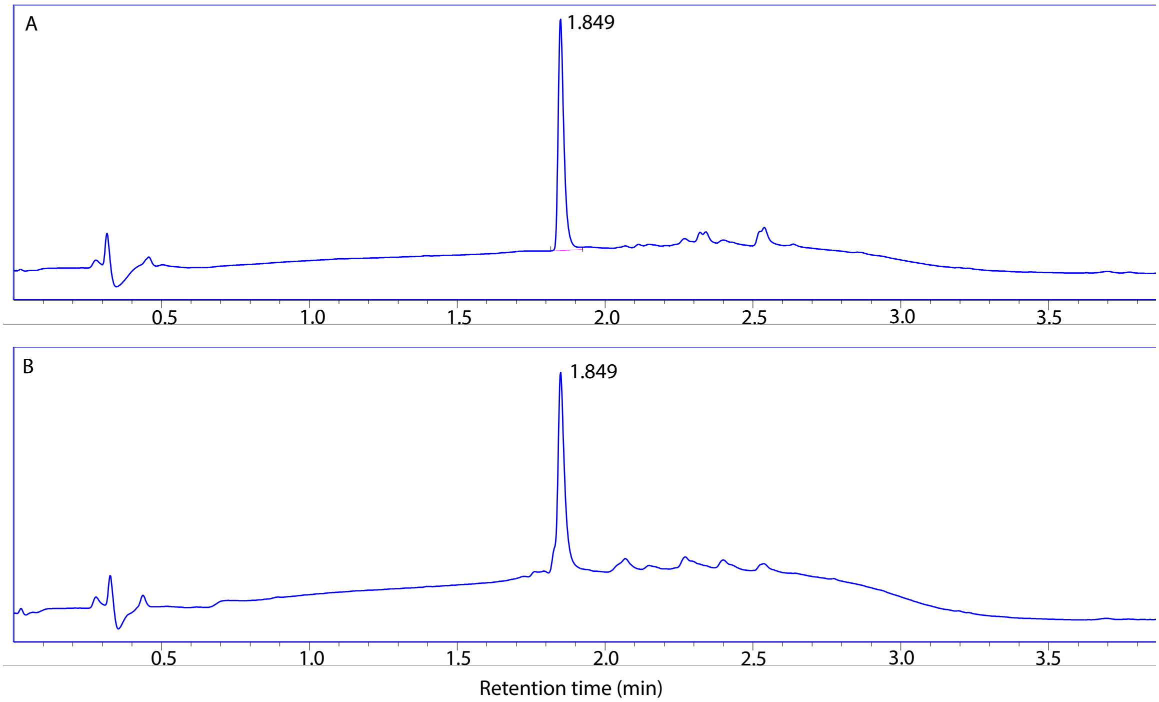

Figure S13. UPLC-DAD (210 nm) chromatograms of (A) natural and (B) synthetic talarolide A.

Mass Spectrum Molecular Formula Report

Analysis Info

Analysis Name	D:\Data\a.salim\Synthetic talarolide A.d	Acquisition Date	10/21/2023 12:45:34 PM
Method	tune-med_AP.m	Operator	a.salim
Sample Name		Instrument / Ser#	micrOTOF 213750.00232
Comment			

Acquisition Parameter

Source Type	ESI	Ion Polarity	Positive	Set Nebulizer	0.8 Bar
Focus	Not active			Set Dry Heater	180 °C
Scan Begin	100 m/z	Set Capillary	4500 V	Set Dry Gas	5.0 l/min
Scan End	1000 m/z	Set End Plate Offset	-500 V	Set Divert Valve	Source

Generate Molecular Formula Parameter

Formula, min.		
Formula, max.		
Measured m/z	Tolerance	Charge
Check Valence	Minimum	Maximum
Nirogen Rule	Electron Configuration	
Filter H/C Ratio	Minimum	Maximum
Estimate Carbon		

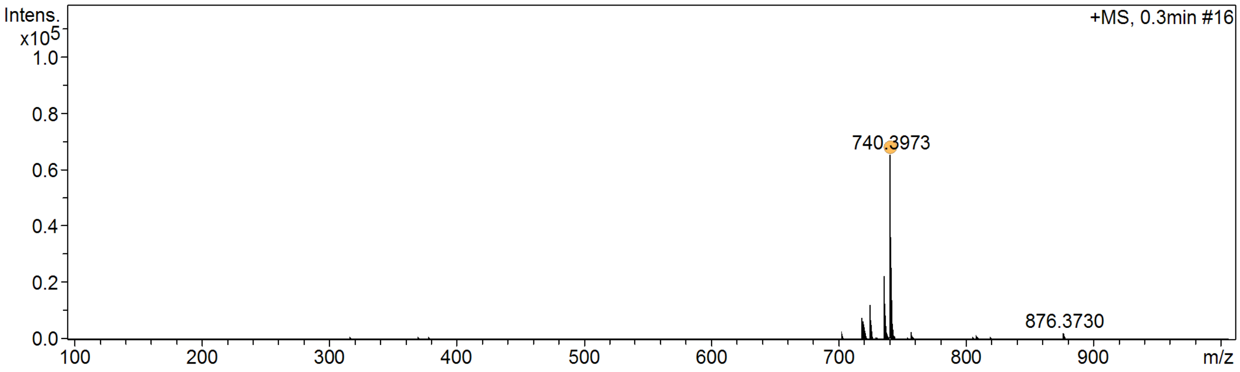

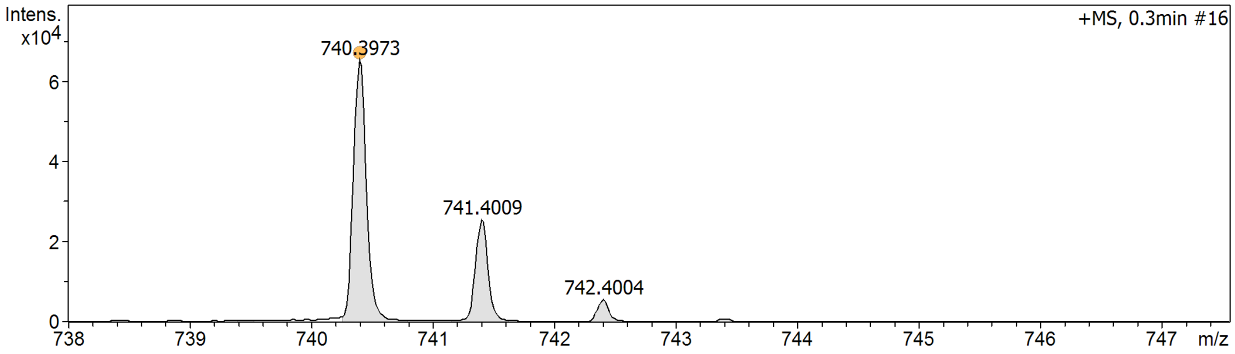

Meas. m/z	#	Ion Formula	m/z	err [ppm]	mSigma	# Sigma	Score	rdb	e⁻ Conf	N-Rule
740.3973	1	C35H55N7NaO9	740.3953	-2.6	14.3	1	100.00	11.5	even	ok

Figure S14. HRMS measurement for synthetic talarolide A (**1**)

3. Characterisation of synthetic intermediates

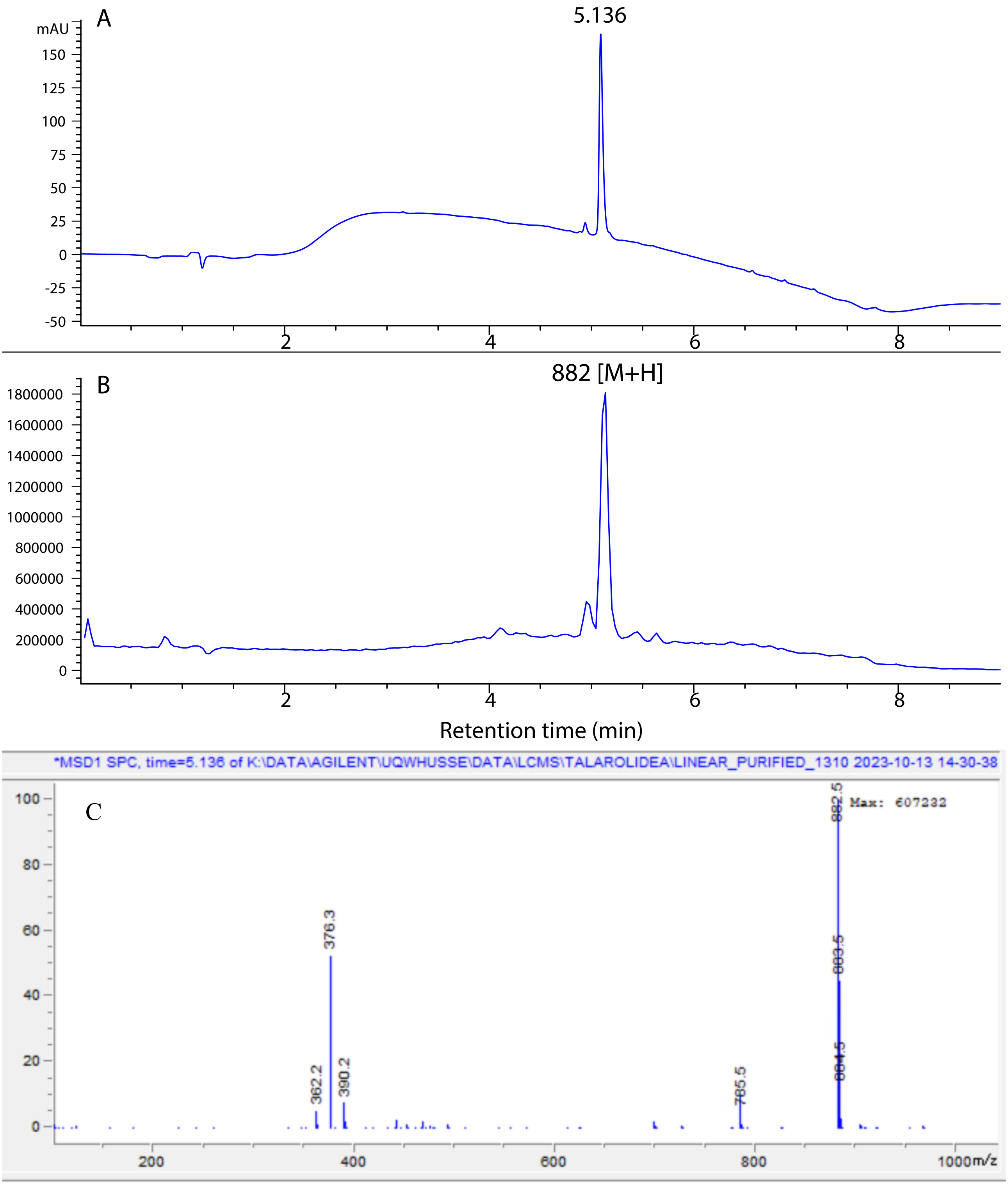

Figure S15. HPLC-DAD-MS chromatograms of protected linear peptide **5**. (A) DAD chomatogram at 210 nm, (B) MS(+) total ion chromatogram, (C) MS spectrum at 5.14 min.

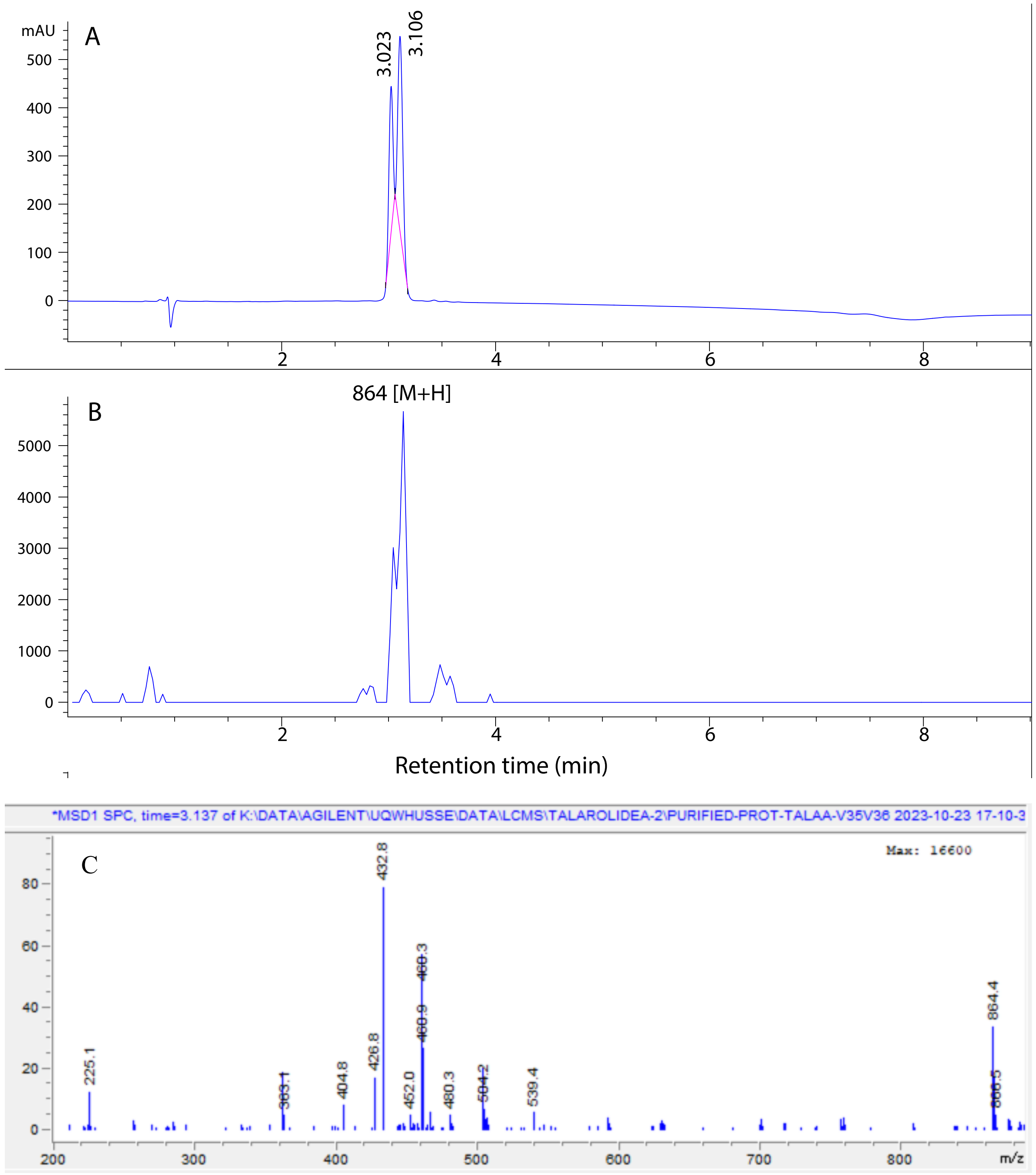

Figure S16. HPLC-DAD-MS chromatograms of protected cyclic peptide **6**. (A) DAD chomatogram at 210 nm, (B) MS(+) extracted ion chromatogram at 864, (C) MS spectrum at 3.13 min.

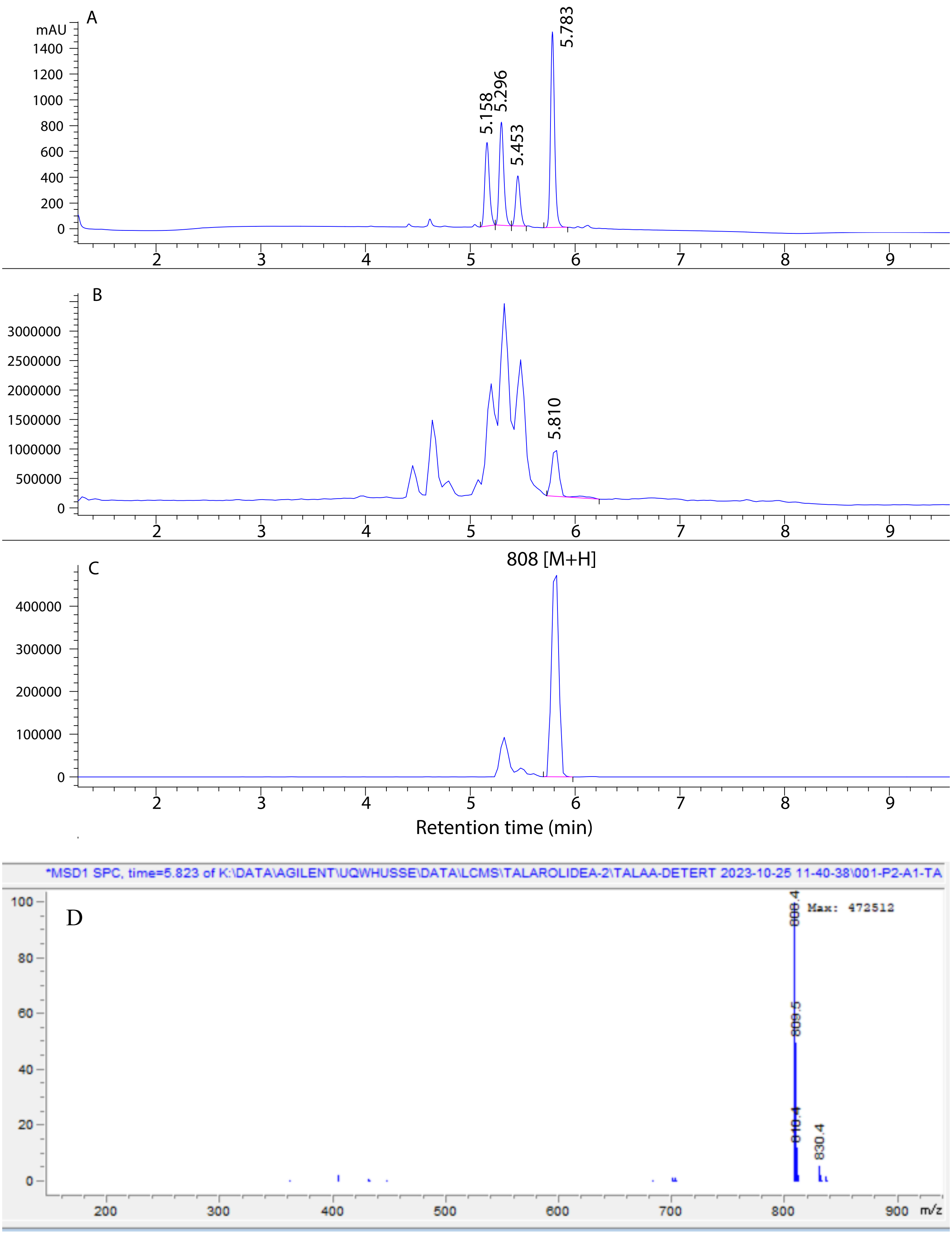

Figure S17. HPLC-DAD-MS chromatograms of crude semi-protected cyclic peptide **7** without *tert* butyl group. (A) DAD chomatogram at 210 nm, (B) MS(+) total ion chromatogram, (C) MS(+) extracted ion chromatogram at 808, (D) MS spectrum at 5.8 min.

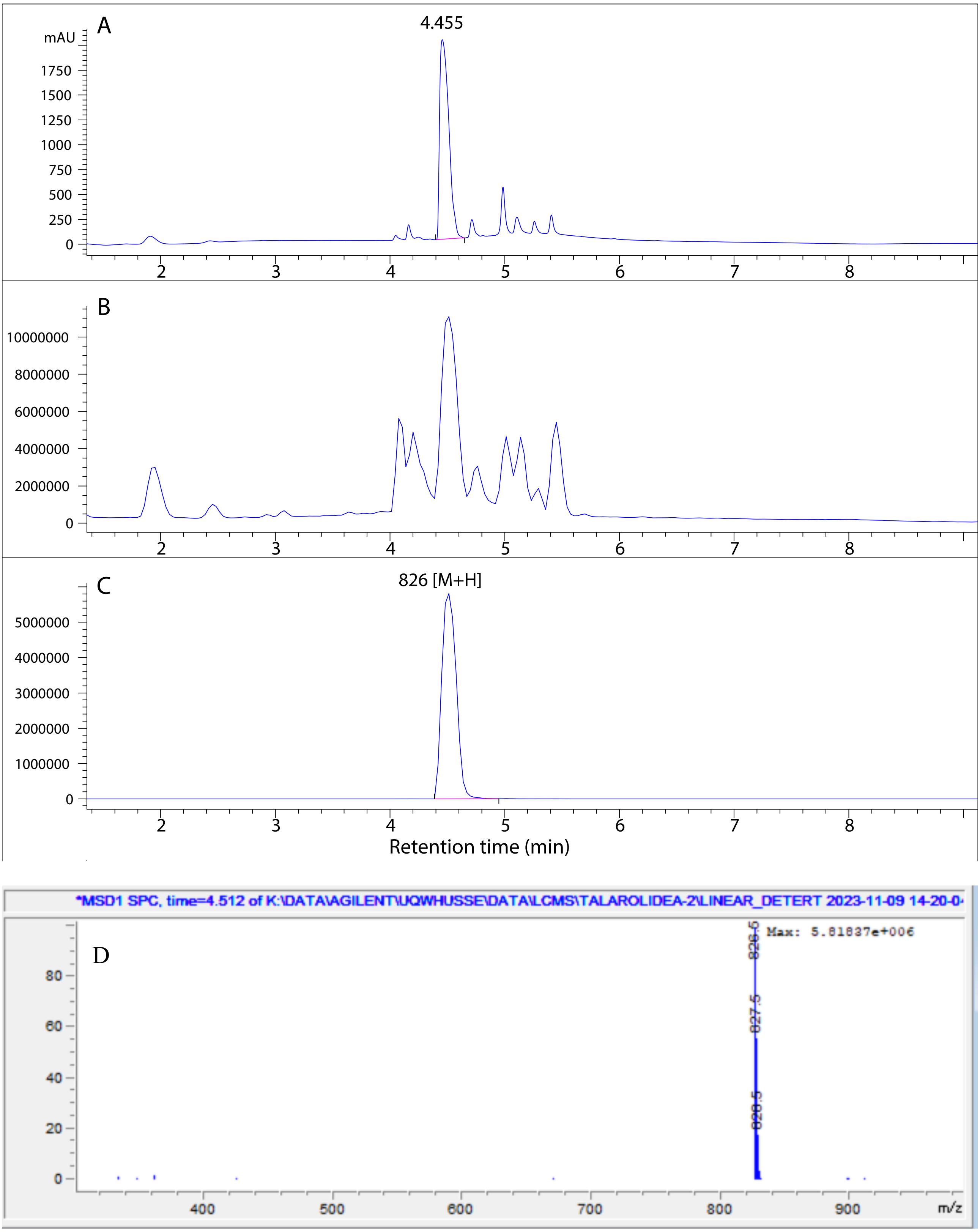

Figure S18. HPLC-DAD-MS chromatograms of crude semi-protected linear peptide **9** without *tert* butyl group. (A) DAD chomatogram at 210 nm, (B) MS(+) total ion chromatogram, (C) MS(+) extracted ion chromatogram at 826, (D) MS spectrum at 4.5 min.

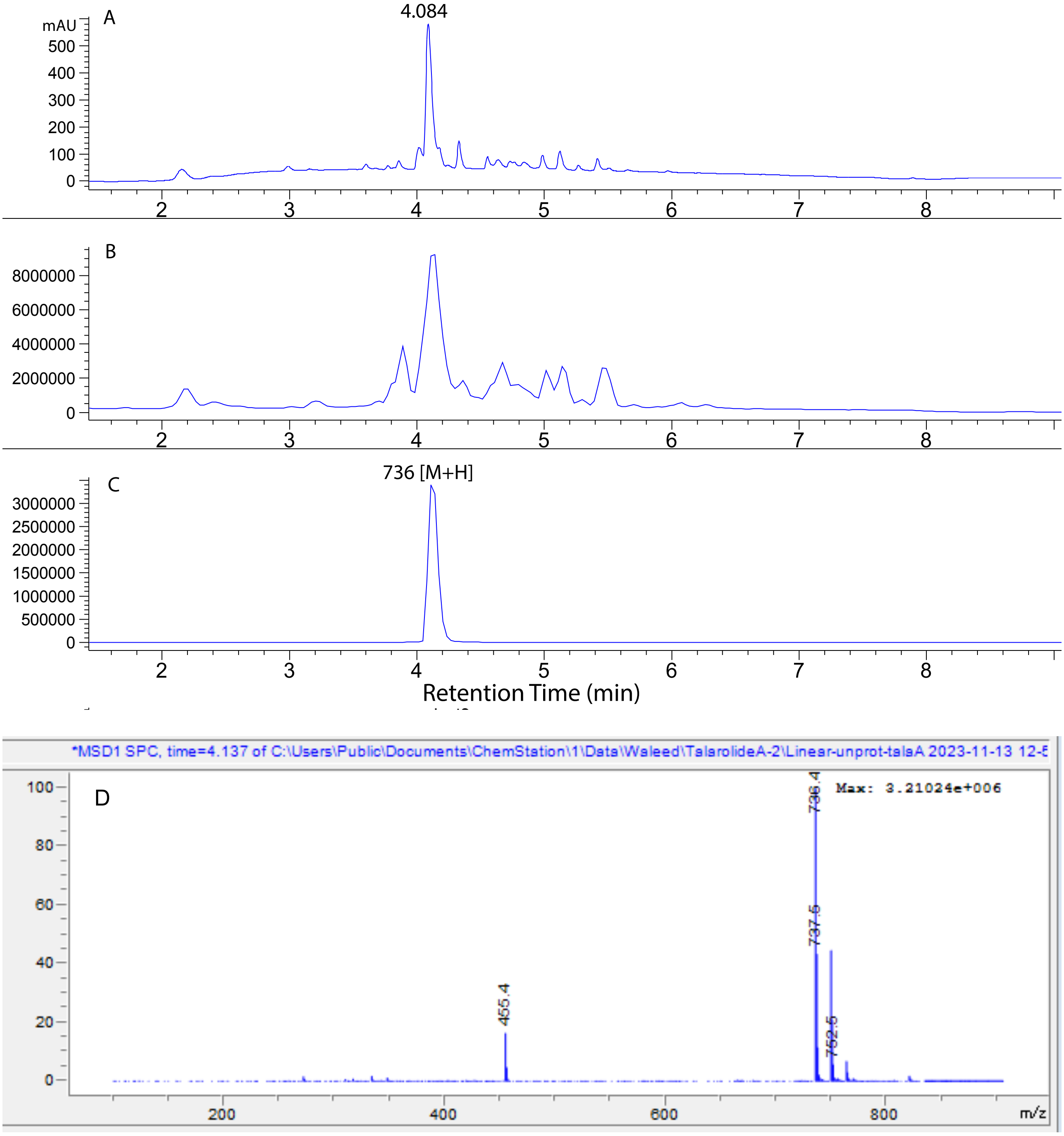

Figure S19. HPLC-DAD-MS chromatograms of unprotected linear peptide **10**. (A) DAD chomatogram at 210 nm, (B) MS(+) total ion chromatogram, (C) MS(+) extracted ion chromatogram at 736, (D) MS spectrum at 4.1 min.
